# Deciphering the Effect of Light Wavelengths in *Monilinia* spp. DHN-Melanin Production and Their Interplay with ROS Metabolism in *M. fructicola*

**DOI:** 10.3390/jof9060653

**Published:** 2023-06-10

**Authors:** Lucía Verde-Yáñez, Josep Usall, Neus Teixidó, Núria Vall-llaura, Rosario Torres

**Affiliations:** IRTA, Postharvest Programme, Edifici Fruitcentre, Parc Agrobiotech Lleida, Parc de Gardeny, 25003 Lleida, Spain; lucia.verde@irta.cat (L.V.-Y.); josep.usall@irta.cat (J.U.); neus.teixido@irta.cat (N.T.)

**Keywords:** abiotic stress, adaptation, black light, blue light, hydrogen peroxide, secondary metabolism, survival

## Abstract

Pathogenic fungi are influenced by many biotic and abiotic factors. Among them, light is a source of information for fungi and also a stress factor that triggers multiple biological responses, including the activation of secondary metabolites, such as the production of melanin pigments. In this study, we analyzed the melanin-like production in in vitro conditions, as well as the expression of all biosynthetic and regulatory genes of the DHN–melanin pathway in the three main *Monilinia* species upon exposure to light conditions (white, black, blue, red, and far-red wavelengths). On the other hand, we analyzed, for the first time, the metabolism related to ROS in *M. fructicola*, through the production of hydrogen peroxide (H_2_O_2_) and the expression of stress-related genes under different light conditions. In general, the results indicated a clear importance of black light on melanin production and expression in *M. laxa* and *M. fructicola*, but not in *M. fructigena*. Regarding ROS-related metabolism in *M. fructicola*, blue light highlighted by inhibiting the expression of many antioxidant genes. Overall, it represents a global description of the effect of light on the regulation of two important secondary mechanisms, essential for the adaptation of the fungus to the environment and its survival.

## 1. Introduction

Light is an important source of information for fungi, an almost unavoidable environmental factor that plays a fundamental role in the behavior of these organisms. Light comprises a wide range of wavelengths, including, among others, ultraviolet and visible light, with its specific wavelengths perceived as different colors. Specifically, many Ascomycota fungi use light to coordinate vegetative growth through the circadian rhythm, establish pathogenic or symbiotic relationships, or even to promote fungal virulence [1,2]. In fact, among the main abiotic signals, light is one of the most influential factors in the *Monilinia* spp. fungus, the main causal agent of brown rot in stone fruit. The response to white light and its different light wavelengths (black, blue, red, and far-red) of the three main species of *Monilinia* (*Monilinia laxa*, *M. fructicola*, and *M. fructigena*) was already demonstrated in terms of pigmentation, growth, conidiation, but also the capacity to infect fruit. These effects were evaluated both at light quality [3] or light intensity [4] levels. At the same time, the fungus also has a plethora of photoreceptors that perceive and react to light signals. In the specific case of the *M. laxa* species, the presence and expression of several photoreceptors has been evaluated, demonstrating its response to direction, light quality and intensity, as well as finally influencing multiple biological responses [5]. 

However, light can also induce detrimental effects in these organisms, including desiccation, osmotic stress, and oxidative stress caused by the overproduction of reactive oxygen species (ROS). The term ROS is defined as a group of partially reduced or excited oxygen molecules, with different chemical reactivities and with a strong oxidizing capacity. These reactive chemical species result from electron transfer reactions that take place mainly in mitochondria, peroxisomes, and as byproducts of the respiratory chain or as a result of enzymatic reactions [6]. In particular, ROS are also produced from oxygen (O_2_) under physiological conditions in response to abiotic and biotic factors. Among the many causes of ROS production, abiotic stress, and particularly light exposure, play a key role. Recent studies in *Botrytis cinerea* have demonstrated that the production of ROS was positively correlated with both ultraviolet radiation (UV) [7] and blue light exposure [8]. To tolerate or alleviate such oxidative stress, fungi have the ability to generate or activate antioxidant machinery, composed of either enzymatic (e.g., superoxide dismutase (SOD), catalase (CAT) and ascorbate peroxidase (APX)), or non-enzymatic compounds (e.g., reduced glutathione (GSH), phenolic compounds, and carotenoids) [9]. 

Hence, a possible mechanism of fungi to avoid or alleviate oxidative stress is through the activation of the secondary metabolism [10]. One of the products derived from such metabolism is melanin, a brown to black heterogeneous polymer derived from phenolic precursors [11]. In general, fungi can synthesize melanin from two different pathways: via the L-3,4-dihydroxyphenylalanine (L-DOPA) pathway, found mainly in basidiomycetes [12] and human pathogens [13], or via the 1,8-dihydroxynaphthalene (DHN) pathway, usually found in ascomycetes [11,14], such as *Monilinia* spp. In this regard, a recent study has demonstrated the presence of DHN–melanin biosynthetic machinery in *M. laxa*, *M. fructicola*, and *M. fructigena* [15] and its regulation under darkness conditions [16]. 

Fungal melanin is unique among biological compounds because it is associated with survival to extreme temperatures and to a wide range of light wavelengths (including ultraviolet), and, hence, it is key in the development and spread of fungal structures [17]. The specific structure of fungal melanin gives rise to its functional properties, including antiradiation, antioxidant, and photoprotective effects, but in the presence of light, it could also cause phototoxic effects, giving rise to a photodamage of phototoxic effects [18]. Thus, melanin production has been shown to protect fungi, such as *B. cinerea* and *Aspergillus* spp. by rapidly absorbing and dissipating ultraviolet (UV) light [13,19,20], although the information is scarce regarding the multiple responses of fungal melanin under the rest of the light wavelengths. On the other hand, there exists no information on the important role of ROS in *Monilinia* species and, especially, their interplay with fungal melanin under different light conditions.

In this study, we have quantified the melanin-like pigments and have described the DHN–melanin biosynthesis in *Monilinia* spp. (*M. laxa*, *M. fructicola*, and *M. fructigena*) upon the exposure to different light wavelengths, as well as its regulation at the molecular level. Furthermore, the ROS-related metabolism under the different light wavelengths has also been unraveled in *M. fructicola*. A relationship between ROS and melanin has also been discussed to further understand the role of these metabolites when *M. fructicola* faces this abiotic stress. 

## 2. Materials and Methods

### 2.1. Fungal Material and Culture Conditions

In this study, three species of *Monilinia* were used: *M. laxa* (ML8L), *M. fructicola* (CPMC6), and *M. fructigena* (GENA6). *Monilinia laxa* and *M. fructicola* were deposited in the Spanish Culture Type Collection (CECT 21100 and CECT 21105, respectively) and the Bioproject code for *M. fructigena* is PRJNA707424.

Fungal suspensions were prepared from 7–9-day-old cultures grown on potato dextrose agar supplemented with 25% tomato pulp (PDA-T). The inoculum of *M. laxa* and *M. fructicola* was obtained, as described by Baró-Montel et al. [21]. Due to the inability of *M. fructigena* to produce conidia, the fungal suspension was prepared, as described by Verde-Yáñez et al. [3]. 

### 2.2. Light and Incubation Conditions 

To study the light effect on the content of melanin-like pigments and ROS-related metabolism, the three *Monilinia* species were exposed to different light conditions: white light (Ta = 5500 K–6000 K, 3350 wl, 400–700 nm) was generated using spectrum Philips LED research modules; black light (UV-A) (370 nm) was generated with 5 fluorescent Philips LED tubes of F20W/T9/BLB; blue light (460 nm), red light (660 nm), and far-red light (740 nm) were generated by using a spectrum Philips LED research Module based on a platform of 9 tubes with 5 LEDs each. 

An incubation period was established with a photoperiod regime of 16-h light and 8-h darkness for 7 days at 20 °C for all light conditions. White light was selected as a control condition.

After the incubation period, fungal suspensions were frozen and ground for the visual inspection of the colony pigmentation, the quantification of melanin-like pigments and hydrogen peroxide (H_2_O_2_), and for gene expression analysis (qPCR). All experiments were performed twice using three replicates per treatment.

### 2.3. Extraction and Quantification of Melanin-like Pigments in Monilinia *spp.* Exposed under Light Conditions

The extraction of melanin-like pigments from *Monilinia* spp. was carried out, as described by Verde-Yáñez et al. [16], using 150 mg of fungal tissue for each species and light condition. The melanin-like content was determined by spectrophotometry (UV-1100, dDBioLab, SLU) at OD_414_, using a standard curve with synthetic melanin (Sigma, St. Louis, MO, USA). The content was expressed as ppm per 100 mg of fresh weight (FW). The experiment was performed twice using three replicates per light condition for each species.

### 2.4. Determination of H_2_O_2_ in M. fructicola Exposed under Light Conditions

The content of H_2_O_2_ levels of *M. fructicola* were determined, as described by Giné-Bordonaba et al. [22], with some modifications. Briefly, 0.5 g of frozen fungal sample was added to 1 mL of 5% trichloroacetic acid (TCA), the samples were homogenized, and then they were centrifuged at 20,000× *g* for 15 min at 4 °C using the PeroxiDetect Kit (Sigma Aldrich, St. Louis, MO, USA) colorimetric assay. Finally, the content of H_2_O_2_ was determined using the FLUOstar Omega (Biogen Científica S.L., Madrid, Spain) microplate reader. The content was expressed as µmol H_2_O_2_ per 100 mg of FW. The experiment was performed twice using three replicates per light condition.

### 2.5. Selection and Identification of Candidate Genes in Monilinia *spp.*

A total of 9 genes involved in the DHN-melanin biosynthetic pathway described by Verde-Yáñez et al. [16] and 11 genes related to oxidative stress were selected for gene expression analysis (Supplementary Table S1). ROS-related genes from *B. cinerea* or *M. laxa* were used as query sequences for a BLAST analysis to search for homologies within the *M. fructicola* (CPMC6) genome [23] using the NCBI Genome Workbench v. 2.11.10 (https://www.ncbi.nlm.nih.gov/tools/gbench/ (accessed on 2 November 2022)), and the BLAST tool implemented therein. The expected value (E) was set to 10^−3^. Identity (>60%) and the fraction of query sequences covered by the matching region (>50%) were used as filter criteria to select only reliable results. The results obtained were verified by performing blastx, blastn, and tblastn analyses.

### 2.6. Fungal RNA Extraction and qPCR Analysis

Fungal RNA was extracted using TRI Reagent^®^ (Sigma, St. Louis, MO, USA), as described by Baró-Montel et al. [21]. A total of 3 biological replicates were used for each species and light conditions. The contaminant DNA was removed by DNase-treatment. Total RNA was quantified using a ND-1000 NanoDrop spectrophotometer (Thermo Scientific, Wilmington, DE, USA). Both RNA integrity and the absence of genomic DNA were assessed by electrophoresis on an agarose gel stained with GelRed^TM^ Nucleic Acid Gel Stain (Biotium, Hayward, CA, USA).

cDNA synthesis was performed on 5 µg of total RNA, using the commercial Superscript IV First-Strand reverse transcriptase cDNA Synthesis Reaction kit (Invitrogen, Carlsbad, CA, USA). 

Gene expression analysis was performed, as described by Baró-Montel et al. [21], using KAPA SYBR^®^ Fast qPCR Master Mix (Kapa Biosystems, Inc., Wilmington, CA, USA) on the 7500 Real Time PCR System (Applied Biosystems, Waltham, MA, USA). Primers used for gene expression analysis (Supplementary Table S2) were designed de novo using the Primer-BLAST tool [24] or retrieved from Verde et al. [16]. *ELONGATION FACTOR 1A* (*EF1-α*) was selected as the reference gene, based on its constant expression among conditions. Primer efficiency was determined using 3-fold cDNA dilutions in triplicate, and primer specificity was checked by analyzing the melting curves at temperatures, ranging from 60 to 95 °C. A non-template control (NTC) was included using water instead of DNA. Relative gene expression was expressed as Mean Normalized Expression (MNE) and calculated using the method described by Muller et al. [25].

### 2.7. Statistical Analysis

All data were collated and subjected to analysis of variance (ANOVA) using JMP^®^ 16 SAS Institute Inc. (v. 16.0.0, Cary, NC, USA.). When the analysis was statistically significant, Tukey’s HSD test was performed at the *p* ≤ 0.05 level for the comparison of means among light wavelengths for each species or among species for each light condition.

## 3. Results and Discussion 

### 3.1. Exposure to Light Wavelengths Induces Different Melanin-like Pigment Productions in Monilinia *spp.*

**Figure 1:**
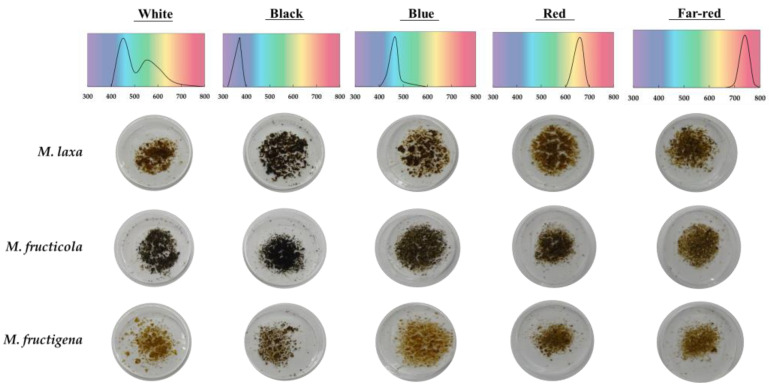
Visual aspect of the different pigmentation of *M. laxa*, *M. fructicola*, and *M. fructigena* grown for seven days under white light or specific light wavelengths (black, blue, red, and far-red). Light spectrum for each light condition is indicated.

The production of melanin-like pigments in *Monilinia* spp. was previously demonstrated under darkness conditions [16]. However, the phenotype and ecophysiology of this fungus largely depends on the light conditions [3]. Herein, we have firstly visually evaluated the different colorations of *Monilinia* spp. grown under white light or under different light wavelengths (black, blue, red, and far-red) (Figure 1). In the case of *M. laxa*, the pigmentation was slightly darker when what was exposed to black light in relation to the rest of the conditions, which induced lighter brown colorations. However, the colonies of *M. fructicola* grown under shorter wavelengths (black and blue) and under white light adopted a black color, while the exposure to long wavelengths (red and far-red) induced a brownish pigmentation. Lastly, for *M. fructigena,* the coloration of the colonies did not differ quite among the different light wavelengths, presenting in general a brown-orange coloration. These differences in the pigmentation pattern were also inferred from our recent study [3] based on the morphological characterization of *Monilinia* spp., in which we showed a different colony phenotype, depending on the light in which the fungus was grown. 

In general, we observed that the colorations obtained for *M. laxa* and *M. fructicola* were more similar than those for *M. fructigena*, which always displayed a lighter pigmentation of the colonies in all the conditions analyzed. These differences in coloration among species could point out to a different pigment accumulation induced by light and/or to the formation of different reproductive structures (i.e., induction of conidiation by the different light exposure). In a study carried out with *B. cinerea* [13], it was demonstrated that both conidia and mycelium accumulate DHN-melanin, although they present a different coloration, being greyish-brown for conidia and black for mycelium. According to this, in a previous study, it was demonstrated that there exists a low capacity of *M. fructigena* to produce conidia [3]. Hence, the mycelium, as the main structure in this species, could now explain the different coloration observed compared to the other species. In this regard, the darker coloration of both *M. laxa* and *M. fructicola* colonies could point to a greater accumulation of pigments in the conidia structures, which is also very closely related to light wavelengths.

Another possibility to explain the brown coloration obtained in the three *Monilinia* species, particularly in blue, red, and far-red light wavelengths (Figure 1) could also be due to the activation of other pathways related to the synthesis of different pigments. In fact, fungi have the ability to synthesize different types of biological pigments, ranging from monomeric (i.e., luciferin, carotenoids, flavonoids, and chlorophylls) to polymeric (i.e., melanin, tannins, and humic substances) [26]. Accordingly, many Ascomycota have those genes necessary for synthetizing both DHN-melanin and carotenoids, thus giving rise to black or brown, as well as orange or red pigments [7]. Besides, the synthesis of these pigments could be related to light, since several studies have shown that the blue light wavelength is able to activate the carotenoid pathway in *Gibberella fujikuroi* [27] and in *Neurospora crasa* [28], which present an orange-brown colony coloration. Therefore, the black colorations could be more associated with melanin, and the brown ones could be associated, for instance, with the synthesis of carotenoids. Apart from carotenoids, there are other pigments, such as flavonoids, activated in response to stress in the fungus *Schizophyllum commune* [29] and under blue light wavelength in *Penicillium digitatum* [30], which provide a white-yellow coloration. Hence, there is a close relationship between light and pigments. In fact, the different coloration degrees could be partly related to the different necessity of *Monilinia* spp. to biosynthesize melanin under these light conditions as a strategy to overcome these stressful conditions.

**Figure 2:**
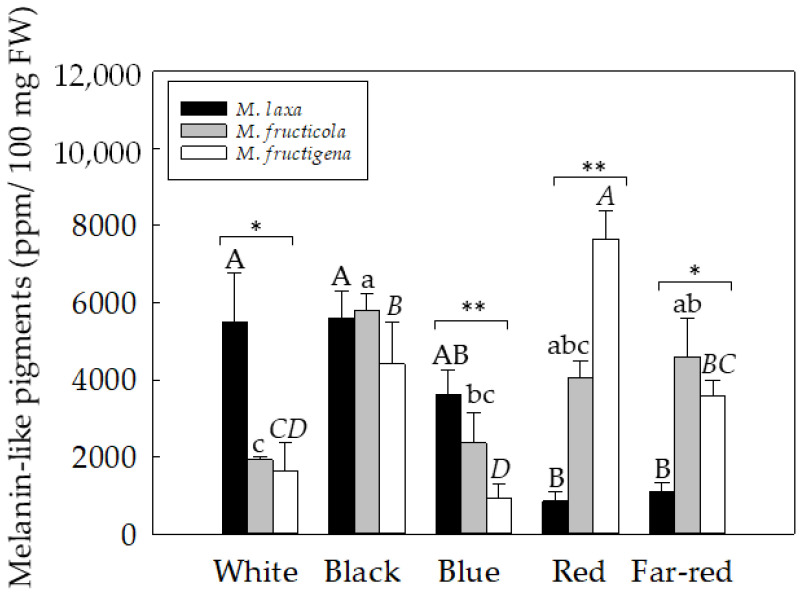
Spectrophotometric quantification (OD_414_) of melanin-like pigments in *M. laxa*, *M. fructicola*, and *M. fructigena* grown on PDA-T medium and incubated for seven days under white light or under specific light wavelengths (black, blue, red, and far-red). Different uppercase (*M. laxa*), lowercase (*M. fructicola*), and italic lowercase (*M. fructigena*) letters indicate significant differences (*p* ≤ 0.05) among the different light wavelengths. For each light condition, asterisks indicate significant differences among species (* *p* < 0.05; ** *p* < 0.01). The error bars represent the standard deviation of the means (n = at least 4).

Thus said, a spectrophotometry analysis was performed to assess the melanin-like pigment content of *Monilinia* spp. grown under white light or under specific light wavelengths for seven days. Results showed significant differences in the production of melanin-like pigment among light conditions, but also among species (Figure 2). In particular, *M. laxa* showed significantly higher melanin-like pigment production when exposed to white and black light compared to long light wavelengths (red and far-red). However, *M. fructicola* showed greater melanin-like pigment production when exposed to black light wavelength (3.03-fold higher) compared to white light. In fact, these results corroborated the darker phenotype of the colony for both *M. laxa* and *M. fructicola* (Figure 1) obtained when grown under the black light. In contrast, under blue light wavelength for *M. laxa*, no significant differences were observed in relation to the other light conditions. The same was observed for *M. fructicola* when it was exposed under red light. Overall, these results are in line with those described for many organisms, in which protection of fungi against UV radiation and its associated stress is provided by pigments, such as melanin, accumulated into the cell wall or plasma membrane [7]. Finally, in *M. fructigena*, a significantly higher melanin-like pigment production appeared when grown under red light wavelength compared to the other light conditions. On the other hand, the low production observed under blue light could be due to a higher production of other pigments, such as carotenoids, which could give rise to a brown-orange coloration, as observed in the pigmentation of the colonies (Figure 1). These results are in line with the study performed in *G. fujikuroi* [27] and in *N. crasa* [28], in which it was observed that the blue light wavelength acts as a signal, triggering the positive regulation of the carotenoid pathway. Therefore, in *Monilinia* spp., each light could activate different pathways, depending on the species.

Overall, it demonstrates that the different *Monilinia* species respond differently to the light wavelength, triggering a different stress response and likely leading to a different fitness and survival under these conditions for each *Monilinia* species. 

### 3.2. The Activation of the DHN-Melanin Biosynthetic Machinery Is Dependent for Each Monilinia *spp.* on the Light Condition

**Figure 3:**
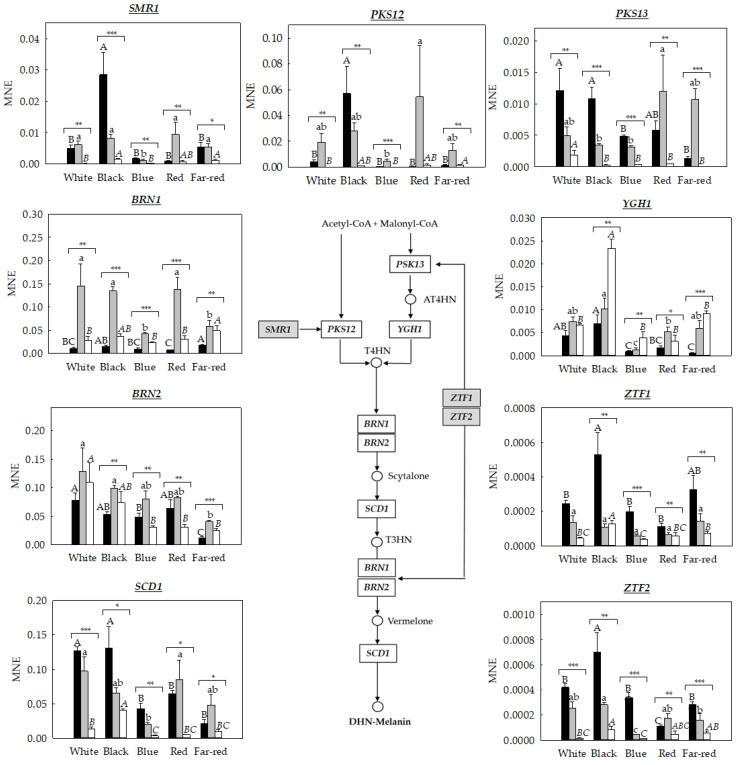
Mean normalized expression (MNE) of DHN–melanin biosynthetic genes of *M. laxa* (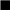), *M. fructicola* (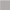), and *M. fructigena* (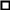) grown for seven days on PDA-T under white light or under specific light wavelengths (black, blue, red, and far-red). Different uppercase, lowercase, and italic letters indicate significant differences (*p* ≤ 0.05) among the different light wavelengths for *M. laxa*, *M. fructicola*, and *M. fructigena*, respectively. For each light condition, asterisks indicate significant differences (* *p* < 0.05; ** *p* < 0.01; *** *p* < 0.001) among species for each light condition. Error bars represent the standard deviation of the means (n = 3). The DHN–melanin pathway is represented. Gene names are specified in white boxes, and the transcription factors are indicated in grey boxes.

The expression of nine genes related to the DHN–melanin pathway, previously identified in the genome of the three main species of *Monilinia* spp. [16], were analyzed in *M. laxa*, *M. fructicola*, and *M. fructigena* under different light conditions (Figure 3).

The DHN pathway is regulated through two initiator genes, *PKS12* and *PKS13*, which are linked to the transcription factors *SMR1*, *ZTF1*, and *ZTF2*. In particular, both *ZTF1* and *ZTF2* are located upstream of *PKS13* and *BRN1* genes in all three *Monilinia* species, whereas *SMR1* is structurally linked with *PKS12* in *M. laxa* and *M. fructicola*, but not in *M. fructigena* [15,16]. 

In other organisms, such as *B. cinerea*, the regulation and initiation of this pathway (through either *PKS12* or *PK13* genes) depends on the presence of light [13]. Thus, in the presence of light, *B. cinerea* activates the pathway through the *PKS13* initiator gene, producing conidiophores and conidia, while, under darkness conditions, the initiator is the *PKS12* gene, involved in the generation of mycelial structures [13]. In contrast, the results obtained for *Monilinia* spp. point out that it is capable of activating both pathways (*PKS12* and *PKS13*) for melanin production, both under darkness condition [16] and different light wavelengths, indicating that light is not a key factor in determining the specific pathway, as occurs in *B. cinerea* [13].

According to these results, we inspected the regulation of the biosynthetic DHN–melanin pathway of *Monilinia* spp. under white light and specific light wavelengths (Figure 3). Among the transcription factors, results demonstrated a higher activation of *SMR1* gene in all three species of *Monilinia* compared to the transcription factors *ZTF1* and *ZTF2*, irrespective of the light condition. However, and in general, all the transcription factors were highly expressed under black light, particularly in *M. laxa*, coinciding with one of the light conditions that produced the highest melanin-like pigment content (Figure 2). The higher expression and production of DHN-melanin under black light conditions could be related to a protection mechanism under this condition. This pattern was similar to that observed in the microorganism *Bipolaris oryzae*, in which the melanin biosynthetic genes increased almost four times under black light [31].

In line with the expression of the transcription factors, *PKS12* and *PKS13* genes were more highly expressed in *M. laxa* and *M. fructicola* than in *M. fructigena*, irrespective of the light condition. Besides, the highest expression in *M. laxa* occurred under exposure to black light for the *PKS12* gene and under black and white light for the *PKS13* gene, also coinciding with the highest melanin-like pigment production in this species (Figure 2). In contrast, in *M. fructicola*, a higher expression of *PKS12* (12.28-fold) and *PKS13* (3.83-fold) genes was observed under red light wavelength compared to blue light wavelength, which was the condition with the lowest expression observed for both genes. These results are in line with the low melanin-like pigment production when *M. fructicola* was exposed under blue light wavelength (Figure 2). For *M. fructigena*, the expression levels of *PKS12* and *PKS13* genes did not differ much, and only the *PKS13* gene was significantly induced under white light exposure. In this species, and differently to *M. laxa* and *M. fructicola*, light seemed to specifically induce the activation of the *PKS13* pathway. Hence, and contrary to what observed in *M. fructigena* under darkness conditions [16], the *PKS13* gene was highly expressed compared to the *PKS12* gene. In this line, the *YGH1* gene, putatively acting downstream the *PKS13* gene, was significantly more activated in *M. fructigena* than in the other species, specially under black light, thus highlighting the *PKS13*-mediated pathway as the main pathway used by this species under lighting conditions. 

Regarding the downstream genes of the DHN pathway, the THN reductase genes (*BRN1* and *BRN2*), which act at two different points during the biosynthesis, were also differentially expressed under the different light wavelengths for both *M. laxa* and *M. fructigena*. In the case of *M. laxa*, the higher *BRN1* gene expression was observed under far-red light wavelength compared to white, blue, and red-light wavelengths. On the other hand, *M. fructicola* was the species that expressed greater levels of the *BRN1* gene, especially under white, black, and red lights in relation to the rest of the light conditions. Contrarily, a significant repression of the *BRN2* gene occurred for the three species under far-red light compared to white light. These results could indicate that *BRN1* and *BRN2* genes act differently and could be even differentially regulated, depending on the lighting condition. Finally, the *SCD1* gene, involved in the last step of the DHN-pathway, was observed to increase in relation to the gene expression in *M. laxa* under white and black light compared to the other light conditions, in line with the results of the melanin-like pigment production in this species, where the white and black conditions were also highlighted for their higher melanin production compared to the rest of the conditions (Figure 2). In the case of *M. fructicola* and *M. fructigena*, it was observed that the lowest expression for the *SCD1* gene occurred under a blue light wavelength, in line with the low melanin-like pigment production in this condition for both species (Figure 2).

Overall, the regulation of the DHN-related genes was light-dependent, but this dependency was species-specific. In general, the blue light wavelength was highlighted for the repression of the expression of genes related to DHN–melanin, which was, in general, in line with the lowest melanin-like production under this condition. These results raise the possibility that blue light could produce a different response to the fungus, inhibiting the activation of some secondary metabolites of the *Monilinia* spp., and, particularly, melanin metabolism and production, which could finally lead to a decreased fitness and impaired survival of the fungus. On the other hand, the black light was the one that most induced the expression of DHN–melanin, especially in *M. laxa* and *M. fructicola*, probably as a strategy of the fungus to protect itself against ultraviolet and its derived oxidative stress [32]. This result was supported by the higher production of melanin-like pigments obtained under black light in *M. laxa* and *M. fructicola* (Figure 2), showing a similar pattern, but different from that obtained for *M. fructigena*. Among the multiple reasons that could explain these differences among species under black light, one of them could be related with photoreceptors. A possible different ability among *Monilinia* species to express those photoreceptors involved in perceiving black light could also explain the different responses observed among species. Another possibility could be that black light induces, in *M. fructigena*, the synthesis of other pigments different from melanin, such as carotenoids, which could also act as antioxidant molecules, protecting the fungus from photodamage induced by light. Finally, another explanation arises in relation to the above-mentioned explanation; in *M. fructigena*, the role of melanin is not as relevant as that in *M. laxa* and *M. fructicola*. This hypothesis could be supported by the low gene expression levels obtained in most of the genes of the DHN–melanin pathway in *M. fructigena* in relation to *M. laxa* and *M. fructicola* (Figure 3).

Accordingly, and given the putative relation between light exposure, melanin, and ROS, we further analyzed the ROS-related metabolism upon exposure to the different light conditions in the *M. fructicola* species. 

### 3.3. ROS-Related Metabolism Is Differently Activated upon Exposure to the Different Light Conditions in M. fructicola

To avoid an oxidative stress situation and ensure its proper functioning, pathogens are able to activate certain secondary metabolisms in response to abiotic stresses, such as light. One of these secondary metabolisms activated by the fungus could be the melanin biosynthetic pathway. Melanin pigment has the biological property to accept and neutralize exogenous free radicals, becoming a powerful antioxidant in stressful situations [26]. In this line, some studies have demonstrated that fungal melanin activates a photoprotective response, protecting the pathogen from the adverse environment [17], and thus preventing the overproduction of ROS, such as H_2_O_2_ [33], which could lead to cell damage and hinder cell survival [34]. However, it has been described that this protection is also dependent on the melanin structure, since eumelanin is capable of triggering a photoprotective activity, but pheomelanin could present phototoxic activity [18] due to its intrinsic content of quinone/semiquinone radicals [35].

**Figure 4:**
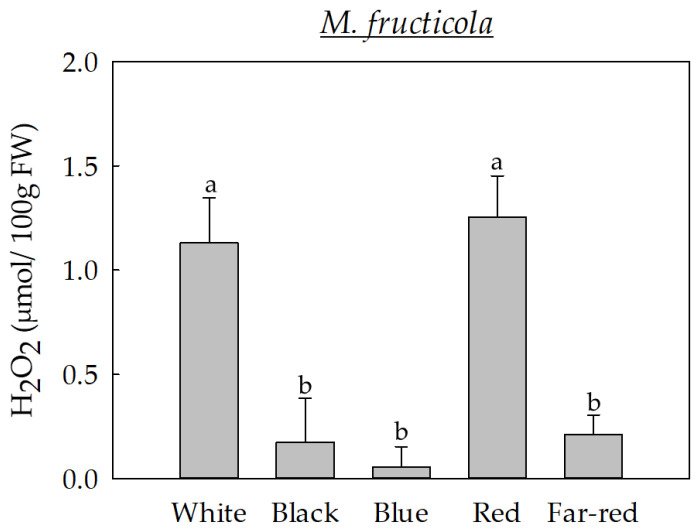
Production of hydrogen peroxide (H_2_O_2_) in *M. fructicola* grown for seven days under white light or under specific light wavelengths (black, blue, red, and far-red). Letters indicate significant differences (*p* ≤ 0.05) among light conditions. Error bars represent the standard deviation of the means (n = at least 4).

Attending to this possible relation between melanin and ROS, in this study, we have selected *M. fructicola*, the most predominant and aggressive species of *Monilinia* to evaluate the ROS-related metabolism after exposure to the different light conditions. Hence, we quantified the amount of H_2_O_2_ in *M. fructicola* after seven days of growing under different light conditions (Figure 4). 

Overall, results indicated that the light wavelengths that induced the greatest production of H_2_O_2_ in *M. fructicola* were white and red light. Specifically, a significantly high H_2_O_2_ production occurred when the fungus was exposed under the red-light wavelength compared to far-red (5.92-fold), black (7.26-fold), and blue (22.43-fold) light wavelengths. In the same vein, a significantly high production was also observed for white light compared to far-red (5.33-fold), black (6.53-fold), and blue (20.18-fold) light wavelengths. These results indicate that both red and white lights induced a different response to the fungus compared to the other light conditions, leading to increased H_2_O_2_ production. The light dependency of the H_2_O_2_ production was also previously documented in *Sclerotium rolfsii*, which displayed a higher H_2_O_2_ production after exposure to 12-h light and 12-h darkness compared to a full darkness condition [36]. 

The higher levels of H_2_O_2_ obtained under white light exposure coincided with the decreased levels in melanin-like pigments in this condition, while the reduced H_2_O_2_ levels observed in some specific lights partly coincided with increased melanin-like pigment production. Hence, these results could, in part, explain the important role of melanin as an antioxidant molecule. However, the different H_2_O_2_ profile observed under the different light conditions could also be related to a different activation of the antioxidant enzymatic machinery mediated by light.

Therefore, to better understand the ROS-related metabolism under different light wavelengths, several genes related to oxidative stress were also analyzed in *M. fructicola* after exposure to several light wavelengths.

### 3.4. The Antioxidant Metabolism of M. fructicola Is Impaired upon Light Exposure

**Figure 5:**
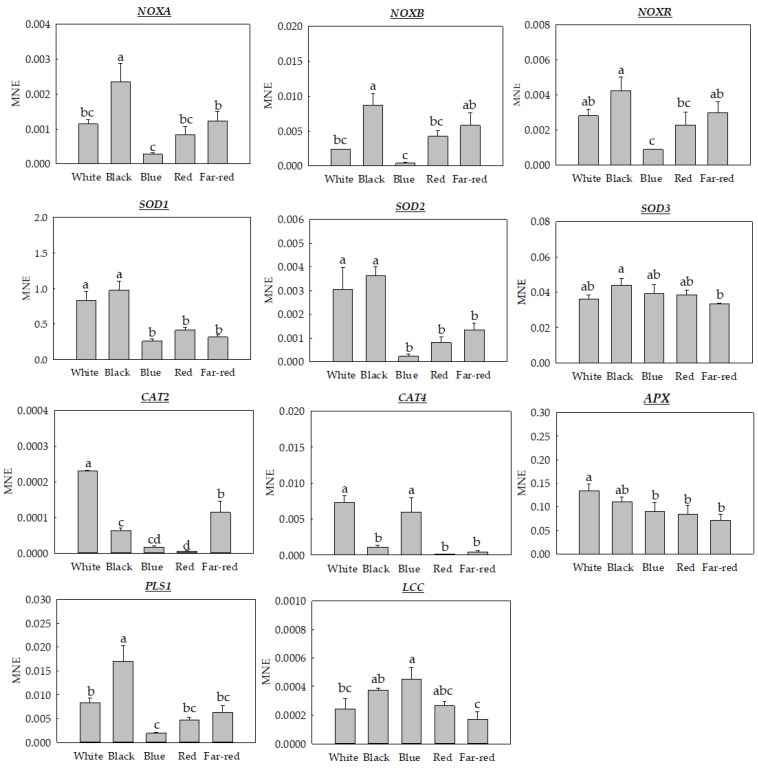
Mean normalized expression (MNE) of oxidative stress related genes in *M. fructicola* grown for seven days on PDA-T under white light or under specific light wavelengths (black, blue, red, and far-red). Letters indicate significant differences (*p* ≤ 0.05) among the different light wavelengths for candidate genes in *M. fructicola*. Error bars represent the standard deviation of the means (n = 3).

As previously discussed, H_2_O_2_ could arise from multiple sources, and a plethora of enzymes act to avoid an oxidative stress situation that can impair or ultimately affect the viability of cells. Due to the possible interplay among melanin, light, and ROS, we evaluated, for the first time, in *M. fructicola*, the gene expression of the main enzymes involved in ROS metabolism and their regulation by light conditions (Figure 5).

Accordingly, 11 genes were identified in the genome of *M. fructicola* (Supplementary Table S1), and their expression was analyzed by qPCR.

The first group of ROS-related genes studied was the NADPH oxidase (NOX) complex, and, specifically, its subunits *NOXA*, *NOXB*, and *NOXR*, which are involved in the regulation of several cellular processes, such as defense, growth, and signaling [6]. The NOX complex and *TETRASPANIN* (*PLS1*) gene [37], with similar functions to *NOXB*, generate O_2_^−^ as a signal for the fungus itself to activate various antioxidant genes, and also as a virulence strategy, generating an oxidative burst when infecting the host [38]. In general, the results obtained for the three NOXs complexes showed a very similar gene expression pattern, obtaining a greater gene expression of *NOXA*, *NOXB*, and *NOXR* genes under black light and the least expression levels under blue light wavelength (Figure 5). Hence, black light induced 8.52-fold, 21.63-fold, and 4.72-fold higher expression levels than blue light wavelength for *NOXA*, *NOXB*, and *NOXR*, respectively. In the same vein, the results for the *PLS1* gene also had the same pattern as the NOXs subunits, showing a higher expression (8.75-fold) under the black light compared to the blue light wavelength. This similar expression patterns could be, in part, explained because of its relationship with the *NOXB* gene. The *PLS1* gene is functionally characterized in *B. cinerea*, and the generation of mutants demonstrated a possible overlapping phenotype [37]. Therefore, the gene expression results obtained under the blue light wavelength for the NOX complex and *PLS1* were always the lowest compared to the other light conditions, probably indicating that blue light could inactivate the expression of this complex.

The action of the NOX complex generates O_2_^−^, which, in turn, triggers a signal that activates SODs [38]. At the same time, the SOD enzymes detoxify O_2_^−^ through the generation of H_2_O_2_ [39,40]. In this study, we identified and analyzed *SOD1*, *SOD2*, and *SOD3* genes (Figure 5). Both *SOD1* and *SOD2* showed a similar pattern upon exposure to the different light conditions, which were in line with that observed for the NOX complex. Specifically, the white and black light conditions induced significantly higher expression levels than the other light conditions. This could be due to the high expression of the NOX complex that generated a high level of O_2_^−^, thus activating the gene expression of SODs, especially *SOD1* and *SOD2*, for black and white light conditions. In turn, blue light induced the lowest expression for these two genes. However, for the *SOD3* gene, differences in gene expression were only observed between black and far-red light wavelengths, being 1.32-fold higher than the expression in the former condition. 

In turn, the H_2_O_2_ generated by the action of SODs could be detoxified by CATs into O_2_ and H_2_O, or by the action of APX with the presence of ascorbate [34,39]. Accordingly, in this study, we analyzed two CAT isoforms, *CAT2* and *CAT4*, and also one *APX* gene from *M. fructicola* (Figure 5). Expression levels for both *CAT2* and *CAT4* were not very high, especially for the *CAT2* gene, for which a low expression level was observed for all light conditions. However, their expression was dependent on light, since different expression levels were obtained among conditions. All light conditions induced a downregulation of *CAT2* expression, showing the highest expression under white light compared to other light conditions. However, for the *CAT4* gene, expression levels were similar between white and blue light conditions, but they were largely downregulated upon exposure to other lights. In the case of the *APX* gene, a significantly higher expression was obtained when exposed under white light compared to blue, red, and far-red light wavelengths. Finally, the *LCC* gene, an oxidase involved in defending against oxidative stress [41,42], was not inhibited by blue light, but by far-red and white light exposure (Figure 5). Although there exists little evidence on the specific function of laccase, in a recent study, it was observed that the expression of laccase genes in the fungus *Podospora anserina* reduced the sensitivity to H_2_O_2_ [43]. This could be in line with the results obtained under the blue light wavelength, which showed a high expression of *LCC* gene, as well as low H_2_O_2_ production. In general, a similar pattern was observed for almost all ROS-related genes, displaying an increase in their expression upon black light, but a repression under blue light wavelength exposure. Hence, the regulation of the antioxidant system of *M. fructicola* seemed to be dependent on light. In many of the genes, blue light seemed to impair the antioxidant metabolism of these species, which could finally lead to an oxidative stress situation that affects the viability and survival of the pathogen. In this vein, in previous studies, it was shown that blue light could control postharvest apple blue mold caused by *Penicillium expansum* [44] and reduce the growth of *P. digitatum* in citrus fruits [45]. Therefore, blue light could also alter the survival and virulence of *M. fructicola* by repressing its antioxidant system. 

### 3.5. Exposure of M. fructicola to Light Wavelengths Induces a Stress Response, Mediated by Melanin and ROS-Related Metabolism

Light triggers multiple responses in fungi, and specifically, in *Monilinia* spp. However, more information is still required to finally clarify the role of light in *Monilinia* spp. and its underlying mechanisms, leading to the adaptability and survival of this devastating fungus in such abiotic stress conditions.

**Figure 6:**
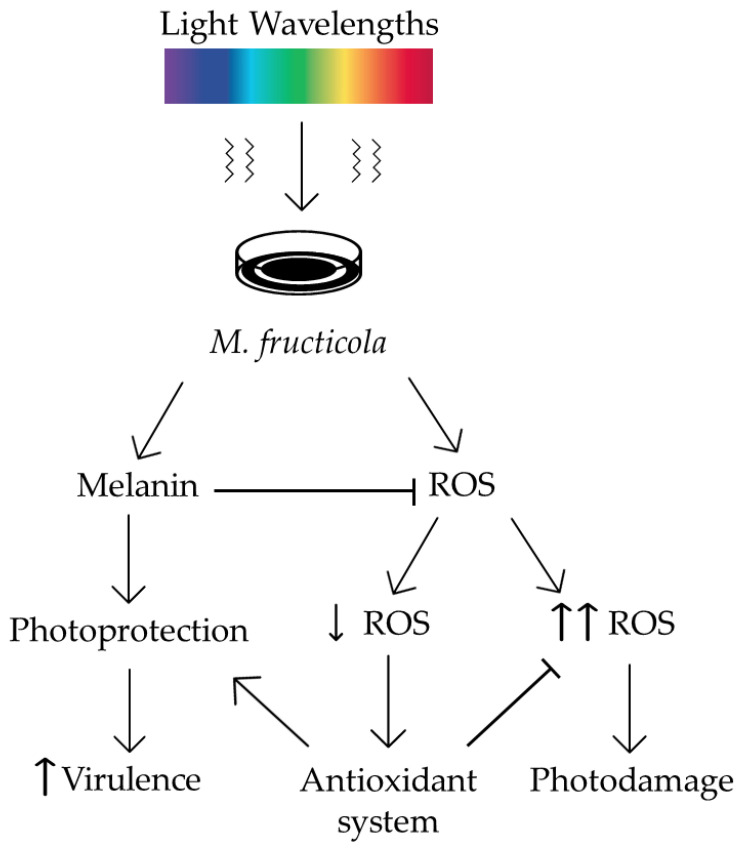
General overview of the behavior of *M. fructicola* upon exposure to light wavelengths and its multiple responses, involving melanin and ROS-related metabolism.

The results obtained in this study shed light on some of the responses that *Monilinia fructicola* triggers upon exposure to white light and its specific light wavelengths (Figure 6). Hence, once *M. fructicola* faces light, and the secondary metabolisms related to melanin and ROS are activated. Melanin could act as an antioxidant molecule, scavenging excess of ROS. However, ROS also act as signaling molecules, activating the antioxidant system of the cell, which, together with the melanin molecule, ensure the photoprotection and the survival and viability of the fungus, probably increasing its virulence. However, light can also induce an overaccumulation of ROS, oxidizing the melanin molecule and producing cytotoxic radicals that could cause photodamage to the cells. In our study, this signaling cascade only took place under specific light wavelengths. 

Thus said, black light exposure promotes the biosynthesis of melanin-like pigments, which finally reduce the levels of ROS, improving the adaptation of *M. fructicola* to this environment and probably enhancing its virulence. On the other hand, this protection disappears upon exposure to blue light, since this light condition alters melanin accumulation and represses all the antioxidant systems of *M. fructicola* involved in host–pathogen interactions (e.g., NADPH oxidases), which could finally impair its virulence and survival.

## 4. Conclusions

The results presented in this study have demonstrated that the DHN–melanin biosynthesis in *Monilinia* spp. is dependent on light, and more specifically, on different light wavelengths. Besides this, the three species responded differently to light. Thus, a similar pattern was observed for both *M. laxa* and *M. fructicola*, highlighting a high gene expression and production of melanin pigments under black light wavelength and a repression under blue light wavelength. For *M. fructigena*, a low production and gene expression was observed after blue light exposure. The ROS metabolism, closely related to melanin, was analyzed for the first time in *M. fructicola*, and results demonstrated its light-dependent regulation. Particularly, blue light was highlighted for inhibiting some of the main antioxidant genes. Hence, all these results contribute to improving knowledge related to the behavior of *Monilinia* spp. upon exposure to different abiotic stress situations and, more particularly, they provide new evidence for the different survival and adaptation capacities of the different species, which will allow a better and targeted control of this pathogen.

## Data Availability

Not applicable.

## Supplementary Materials

The following supporting information can be downloaded at: https://www.mdpi.com/article/10.3390/jof9060653/s1, Table S1: DHN-melanin related genes from *Monilinia* spp., and ROS-related genes from *M. fructicola*. Melanin genes were retrieved from our previous study [16]. Genes related to ROS were identified in the *M. fructicola* genome after a BLAST analysis using genes from *B. cinerea* and *M. laxa* as query sequences. Gene names and their corresponding gene IDs are specified. Table S2: Real-time PCR primer set (including target gene, primer name, primer sequence, and its source) to assess the expression pattern of the selected genes.
