# Supplementary material for: Deciphering the Effect of Light Wavelengths in Monilinia spp. DHN-Melanin Production and Their Interplay with ROS Metabolism in M. fructicola
Source: J Fungi (Basel). 2023 Jun 10;9(6):653. doi: 10.3390/jof9060653 (PMC10304210; doi:10.3390/jof9060653)
Supplement: Supplementary file 1 [file jof-09-00653-s001.zip › Supplementary Table S1.pdf]

Supplementary Table S1. DHN-melanin related genes from *Monilinia* spp., and ROS-related genes from *M. fructicola*. Melanin genes were retrieved from our previous study [16]. Genes related to ROS were identified in the *M. fructicola* genome after a BLAST analysis using genes from *B. cinerea* and *M. laxa* as query sequences. Gene names and their corresponding gene IDs are specified.

Gene	Full gene name	ID Query sequence	ID Query sequence
PKS12	*POLYKETIDE SYNTHASE- 12*	Bcin02g08770	*M. laxa:* Monilinia__049320<br>*M. fructicola:* MFRU_042g00180.1<br>*M. fructigena:* g3016.t1
PKS13	*POLYKETIDE SYNTHASE- 13*	Bcin03g08050	*M. laxa:* Monilinia__009450<br>*M. fructicola:* MFRU_053g00290.1<br>*M. fructigena:* g3467.t1
SMR1	*SCLEROTIAL MELANOGENESIS REGULATORY GENE- 1*	Bcin02g08760.1	*M. laxa:* Monilinia__009400<br>*M. fructicola:* MFRU_053g00340.1<br>*M. fructigena:* g3472.t1
YGH1	*YELLOWISH-GREEN HYDROLASE- 1*	Bcin02g04360.1	*M. laxa:* Monilinia__012010<br>*M. fructicola:* MFRU_030g00600.1<br>*M. fructigena:* g3325.t1
BRN1	*BROWN 1*	Bcin03g08100	*M. laxa:* Monilinia__025870<br>*M. fructicola*: MFRU_016g00660.1<br>*M. fructigena*: g3016.t1
BRN2	*BROWN 2*	Bcin04g04800	*M. laxa:* Monilinia__009410<br>*M. fructicola:* MFRU_053g00330.1<br>*M. fructigena:* g3471.t1
SCD1	*SCYTALONE DEHYDRATASE- 1*	Bcin03g08110.1	*M. laxa:* Monilinia__049320<br>*M. fructicola:* MFRU_042g00180.1<br>*M. fructigena:* g3016.t1
ZTF1	*TRANSCRIPTION FACTOR 1 (CLUSTERED WITH PKS12 OR PKS13)*	Bcin03g08090	*M. laxa:* Monilinia__009420<br>*M. fructicola:* MFRU_053g00320.1<br>*M. fructigena:* g3470.t1
ZTF2	*TRANSCRIPTION FACTOR 2 (CLUSTERED WITH PKS12 OR PKS13)*	Bcin03g08080	*M. laxa:* Monilinia__009430<br>*M. fructicola:* MFRU_053g00310.1<br>*M. fructigena:* g3469.t1
NOX A	*NADPH OXIDASE A*	XP_001550350.1	*M. fructicola*: MFRU_002g00910.1
NOX B	*NADPH OXIDASE B*	XP_024547116.1	*M. fructicola*: MFRU_056g00360.1
NOX R	*NADPH OXIDASE R*	XP_001555495.1	*M. fructicola*: MFRU_027g00750.1
PLS1	*TETRASPANIN*	XP_001551733.2	*M. fructicola*: MFRU_037g00190.1

SOD1	*SUPEROXIDE DISMUTASE 1*	Bcin03g03390	*M. fructicola*: MFRU_003g03910.1
SOD2	*SUPEROXIDE DISMUTASE 2*	Bcin_06g03160	*M. fructicola*: MFRU_005g03090.1
SOD3	*SUPEROXIDE DISMUTASE 3*	Bcin01g03830	*M. fructicola*: MFRU_009g02240.1
CAT2	*CATALASE 2*	Bcin11g06450	*M. fructicola*: MFRU_040g00100.1
CAT4	*CATALASE 4*	Bcin05g00730	*M. fructicola*: MFRU_002g00600.1
APX	*ASCORBATE PEROXIDASE*	Bcin_01g09360	*M. fructicola*: MFRU_021g00350.1
LCC	*MULTICOPPER OXIDASE, LACCASE*	Monilinia__03303	*M. fructicola*: MFRU_009g01200.1
