# Supplementary material for: Deciphering the Effect of Light Wavelengths in Monilinia spp. DHN-Melanin Production and Their Interplay with ROS Metabolism in M. fructicola
Source: J Fungi (Basel). 2023 Jun 10;9(6):653. doi: 10.3390/jof9060653 (PMC10304210; doi:10.3390/jof9060653)
Supplement: Supplementary file 1 [file jof-09-00653-s001.zip › Supplementary Table S2.pdf]

Supplementary Table S2. Real-time PCR primer set (including target gene, primer name, primer sequence, and its source) to assess the expression pattern of the selected genes.

Target gene	Primer name	Primer sequences (5´- 3´)	Source
PKS12	PKS12- Fw<br>PKS12- Rv	TGCTACCAACCACTCAGCAG<br>TTGGGATCAACACCCGCATT	[16]
PKS13	PKS13- Fw<br>PKS13- Rv	GCCTACGAGGCCATGGAAAT<br>TCGATGTCTTGAGCAGCGTT	[16]
SMR1	SMR1- Fw<br>SMR1- Rv	CCATTACCCGGTGGTGTTCC<br>GCATATCTGCTGGCACATGG	[16]
YGH1	YGH1- Fw<br>YGH1- Rv	CGAGCAGCTTGTGTCTATCG<br>TTTTGGGCTTCCCATGCCTT	[16]
BRN1	BRN1- Fw<br>BRN1- Rv	GGATCTGGCCAAGGTATCGG<br>ATGTCCCCTGGGACTGCTAT	[16]
	BRN1- Fw<br>BRN1- Rv	CCGAACCGACATGTACCACA<br>GCGACCAAGTGCAAGCATAC	[16]
BRN2	BRN2- Fw<br>BRN2- Rv	CCAATATGTCCGGCGTACCA<br>AGCGATACCGTTGCAAGTGA	[16]
SCD1	SCD1- Fw<br>SCD1- Rv	GACTACACCACCATCGGCAA<br>CGCCGAGAAGATGTTGGGTT	[16]
ZTF1	ZTF1- Fw<br>ZTF1- Rv	TCCCAACAATGCTCGTCGAAT<br>GGTACGCAGCAGCCATACTC	[16]

ZTF2	ZTF2- Fw<br>ZTF2- Rv	TGTCGAGCTTTGGATGGCTAC<br>TGAGCCATGCGGTACATCTTA	[16]
NOXA	NOX A- Fw<br>NOX A- Rv	GAAGGATGGCGTTGGGAACT<br>TGACGAACGACTCGAACGAT	Vall-llaura, unpublished
NOXB	NOX B- Fw<br>NOX B- Rv	TGGTGCCGGTATTGGTGTTA<br>TCTTGAGCTTCGATTGCGAGT	Vall-llaura, unpublished
NOXR	NOX R- Fw<br>NOX R- Rv	AGATCCGTGAACGTGACGAC<br>TGATTCGGATCTTCGGCTGG	Vall-llaura, unpublished
PLS1	PLS1-Fw<br>PLS1-Rv	CATCTGGTTCGGGACGCTC<br>CACCCCGGCATCGTCG	Vall-llaura, unpublished
SOD1	SOD1-Fw<br>SOD1-Rv	GTGACAACACCAACGGCTGC<br>GGACCTCATCGGTGGGAGCAC	This study
SOD2	SOD2-Fw<br>SOD2-Rv	GCAACCATCCCCGAAGACAA<br>TGTGGGGAGGTTGGAGAATTG	This study
SOD3	SOD3-Fw<br>SOD3-Rv	ATCTAGCAGGTTCGCCATTCC<br>GACGCAAAGAATTGGCGTGA	This study
CAT2	CAT2-Fw<br>CAT2-Rv	ACGCGATGTTCATGGCTTTG<br>CGTCCATGTGACGGAATGAC	This study
CAT4	CAT4-Fw<br>CAT4-Rv	CTCATGGGTGATCGCGGTAT<br>CGTAGTCGGGTGACTTCTCG	This study

APX	APX-Fw<br>APX-Rv	CCAGGTCGTCAAGACAAGGA<br>ATCAGTGTGGCAACGACCAA	This study
LCC	LCC-Fw<br>LCC-Rv	TTCCCTTGGGGATCGTTGAC<br>AAGTGGCCCTGGATATTGCC	This study
EF1-α	EF1-α-Fw<br>EF1-α-Rv	CTGAGTACCCACCTCTCGGA<br>ACCGGCCTTTTCTTGCTTCT	[3]
